# Calling the roll on *Laxus oneistus* immune defense molecules

**DOI:** 10.1007/s13199-012-0157-3

**Published:** 2012-02-25

**Authors:** Silvia Bulgheresi

**Affiliations:** 1 Center of Anatomy and Cell Biology, Laboratories of Genome Dynamics, Medical University of Vienna, Währingerstrasse 10, 1090 Vienna, Austria; 2 Department of Genetics in Ecology, University of Vienna, Althanstrasse 14, 1090 Vienna, Austria

“Say what you see”.

(*The Geographical History of America or the relation of Human Nature to the Human Mind*, Gertrude Stein, 1936)

## *Laxus oneistus*’ identity tag

**Fig. 1:**
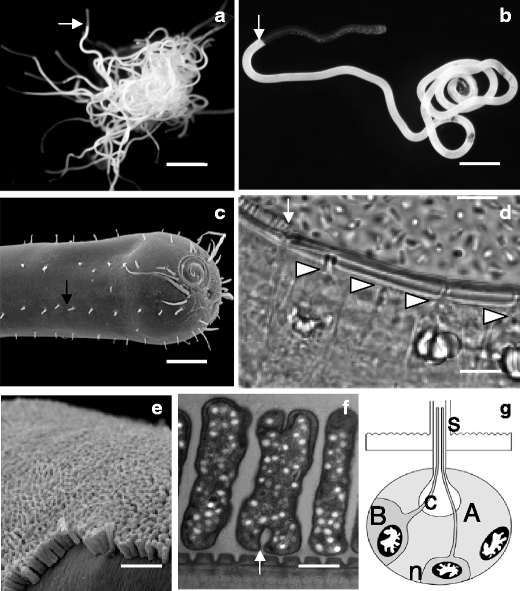
The stilbonematid nematode *L. oneistus*. **a** Light microscope image of an aggregation of approximately 50 living individuals; white arrow points to the beginning of the bacterial coat in one *L. oneistus* individual; scale bar is 2 mm. **b** Light microscope image of a single nematode; white arrow points to the beginning of the bacterial coat; scale bar is 150 μm. **c** Scanning electron microscope image of the anterior cuticle from which numerous setae protrude, black arrow points to one of them; scale bar is 20 μm. **d** Light microscope image of glandular sense organs (GSOs); the arrow points to the beginning of the bacterial coat, the arrowheads point to the canals of four GSOs; scale bar is 10 μm. **e** Scanning electron microscope image of the bacterial coat; scale bar is 3 μm. **f** Transmission electron microscope image of bacterial ectosymbionts; arrow points to one bacterium undergoing binary fission; scale bar is 0.5 μm. **g** Schematic representation of a GSO according to Bauer-Nebelsick et al. 1995 depicting its basic components: the A and B gland cells, the neuronal cell (n), the canal (C) and the seta (s). **a** and **b** are by Ulrich Dirks, **c** by Mario Schimak, **d** by Joerg A. Ott, **e** and **f** by Nikolaus Leisch

**Fig. 2:**
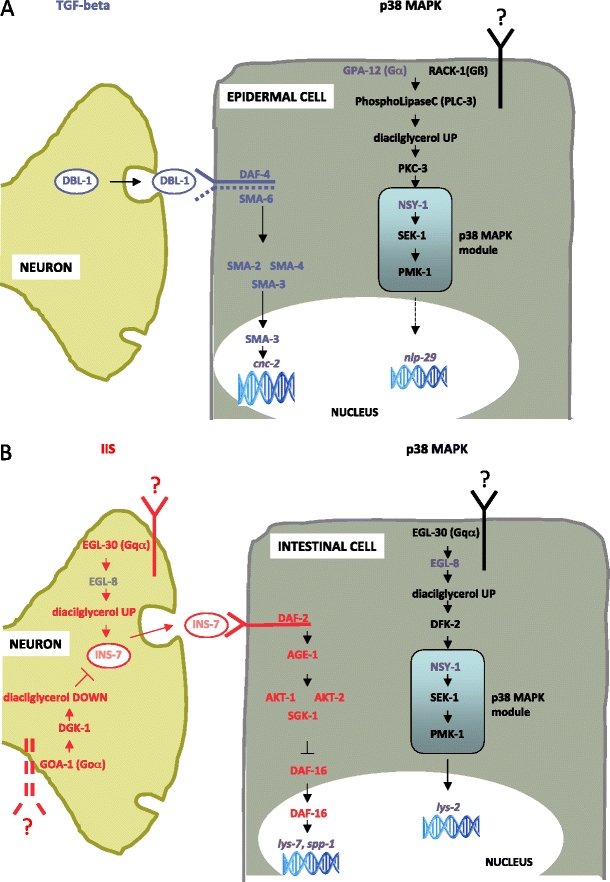
Schematic representation of signaling pathways regulating epithelial immune response in nematode epidermal (**a**) and intestinal (**b**) cells, adapted from Tan and Shapira 2011. The scheme is based on *C. elegans* immune response. GPA-12, NSY-1, cnc-2, lys-2, lys-7, nlp-29, and spp-1 are displayed in grey as transcripts corresponding to *L. oneistus* orthologs have not been identified yet. The p38 MAPK pathway (*black receptors*, *letters*, and *arrows*) regulates the expression of *nlp-29*, an antimicrobial peptide (AMP) encoding gene in the epithelial cell. A second AMP, encoded by *cnc-2*, is regulated by neuronally expressed *dbl-1* that actives the epidermal cell TGF-β pathway (*blue letters*). The G-protein-coupled receptors that engage epithelial Goα signaling to modulate epidermal cell immune response is not known. **b** In the intestinal epithelial cell, the IIS pathway (*red letters*, *receptors* and *arrows*) is regulated primarily by the insulin-like ligand released by the neuron. IIS activity regulates DAF-16 subcellular localization. A separate G-protein signaling pathway (*black receptors*, *letters* and *arrows*) modulates the activity of the p38 MAPK module through phospholipases such as EGL-8, which determines the level of diacylglycerol, and protein kinase D (DFK-2). The G-protein-coupled receptors that engage epithelial Goα and neuronal Goα and Gqα signaling to modulate intestinal cell immune response are not known. See Appendix and Tables 1 and 2 for details

*L. oneistus, named* from the Greek *oneistos* meaning “most useful”, is a marine nematode that occurs in subtidal coarse sand from below the waterline. So far, it has only be collected from the sediment of the barrier reef surrounding the island of Carrie Bow Cay, in Belize (Ott et al. 1995). When extracted from the sediment, it tends to curl around its body or that of other conspecifics, due to its thigmotactic instinct, leaving only its anterior-most part protruding from such tight aggregations (Fig. 1a). 18S rRNA-gene based phylogeny placed *L. oneistus* into a group of closely related genera classified as the *Stilbonematinae* subfamily (Ott et al. 2004a, b). The group is monophyletic and forms a distinct clade within the *Desmodoridae* (Kampfer et al. 1998; Bayer et al. 2009). All the known stilbonematids establish ectosymbioses with thiotrophic Gammaproteobacteria (see below). A single layer of rod-shaped bacteria, in the case of *L. oneistus,* covers the cuticle of the whole nematode, except for an anterior region, which in males can be up to 1/10 of their body length (ca. 1 mm). For simplicity, we refer to the ectosymbiont-free region of the *L. oneistus* body as the anterior (A) region and to the ectosymbiont-associated region as the posterior (P) region. Notably, the cuticle thins at the bacterial-coat onset (Urbancik et al. 1996) (Fig. 2). Besides the bacterial coat, two additional morphological characters unify all stilbonematids: a weak pharynx, glandular rather than muscular (Hoschitz et al. 2001), and unique epidermal organs known as glandular sense organs (GSOs; Fig. 1c, d and g) (Bauer-Nebelsick et al. 1995). Hundreds of GSOs underlie the worm cuticle throughout the A-P axis. They are separated from the cuticle only by an epidermal layer, which is thin and does not possess a basal lamina. Two gland cells (A and B) and a sensory neuron are the basic components of the GSO (Bauer-Nebelsick et al. 1995). This bears a central, pear-shaped canal (Fig. 1c, e and g) where secretory products can accumulate. The canal crosses the epidermis and cuticle and terminates in the pore of a bristle-like structure called the seta (Fig. 1b e and g). Therefore, a continuum exists between the GSO canal and the nematode surface. The P GSOs are crucial to the *L. oneistus* ectosymbiosis because they express and secrete the Ca^2+^-dependent sugar-binding protein Mermaid. This C-Type Lectin Domain-containing protein (CTLD) is mannose-, galactose-, and N-acetylglucosamine-specific (Bulgheresi et al. 2006; Zhang et al. 2006; Nabatov et al. 2008). Moreover, its carbohydrate recognition domain (CRD) is structurally and functionally similar to the CRD of the human dendritic cell-specific immunoreceptor DC-SIGN (Bulgheresi et al. 2006; Zhang et al. 2006; Nabatov et al. 2008; Mittal et al. 2009). The surface of the A cuticle is both free of bacteria and Mermaid lectin. Adding recombinant Mermaid causes *L. oneistus* ectosymbiont to aggregate or to detach from the worm. This suggests an involvement of this CTLD in both symbiont-symbiont and host-symbiont attachment. Different recombinant Mermaid isoforms vary in their affinity for particular stilbonematid symbionts. In particular, DDA-type Mermaids—which possess D, D and A at position 105, 108 and 109, respectively—appear to be the most expressed isoforms in *L. oneistus*. DDA-type Mermaids show a higher affinity for the ectosymbiont of *L. oneistus* than for that coating the co-occuring stilbonematid *Stilbonema majum*. On the other hand, GDA-type Mermaids are expressed by *S. majum* but not by *L. oneistus* and agglutinate the *S. majum* ectosymbiont more efficiently than that of *L. oneistus*. These data suggest that the expression of different Mermaid isoforms may mediate the attachment of a specific ectosymbiont to a given stilbonematid species.

The reason for the P-specific secretion of the lectin Mermaid is unclear. Although no differences among *L. oneistus* A and P GSOs were reported (Bauer-Nebelsick et al. 1995), a recent, more detailed morphological analysis lead to the identification of three types of setae, with the longest type being restricted to the P region (A. Schmidt, unpublished). In another desmodorid nematode (*Desmodora ovigera*) the histochemical properties of the GSOs underlying long setae differs from those of the GSOs underlying short setae. Moreover, both in *D. ovigera* and in *Laxus cosmopolitus* histochmical differences between somatic and sexual GSOs have been found (I. Eichinger, unpublished). Taken together, it is possible that *L. oneistus* bears different GSO types and that these are differently distributed along the worm A-P axis. Consequently, the P-specific Mermaid secretion may reflect the A-P distribution of a specific GSO type. Thorough ultrastructural analysis is needed to clarify this. Moreover, the identification and localization analysis of additional GSO-specific transcripts and proteins will help reveal the function and physiology of these enigmatic organs.

## *L. oneistus* ectosymbiont’s identity tag

The *L. oneistus* ectosymbiont is a 0.6 × 2.1 μm rod aligned perpendicularly to the nematode surface and forms a bacterial layer that resembles a columnar epithelium (Fig. 1e and f). The frequency of dividing bacteria is almost double in the vicinity of bacteria-free areas (Polz et al. 1992). According to 16S-rRNA gene phylogeny, all the characterized stilbonematid ectosymbionts belong to the marine oligochaete and nematode thiotrophic symbiont (MONTS) cluster that includes all gammaproteobacterial sulfur-oxidizers stably associated with these invertebrates (Polz et al. 1994; Bayer et al. 2009; Bulgheresi et al. 2011; Heindl et al. 2011). The MONTS are a basal group of Gammaprotobacteria (Harald R. Gruber-Vodicka, unpublished data) that likely originated from marine free-living bacteria because their closest relatives are the free-living sulfur purple bacteria of the Chromatiaceae (Heindl et al. 2011). Evolutionary transitions from free-living to mutualistic lifestyles are indeed common among bacteria (Sachs et al. 2011). The 16S rRNA-genes of the ectosymbionts of three stilbonematid species PCR amplified from pelagic seawater samples are also part of the MONTS cluster (Heindl et al. 2011). However the *L. oneistus* 16S rRNA-gene has never been amplified from the marine environment (unpublished data). In addition to their 16S rRNA-gene based phylogenetic placement, uptake of ^14^C bicarbonate (Schiemer et al. 1990) and the presence of RuBisCo enzymatic activity indicate autotrophy [(Polz et al. 1992). Note that *L. oneistus* was referred to as *Catanema* sp. in publications predating Ott et al. (1995)]. Enzymatic activity of ATP sulfurylase and sulfite oxidase as well as the presence of elemental sulfur in symbiotic but not in aposymbiotic *L. oneistus* (Polz et al. 1992) and the cloning of the alpha subunit of the adenosine-5′-phosphosulfate (APS) reductase gene (*apr*A; (Bayer et al. 2009)) support sulfur-oxidation by the *L. oneistus* ectosymbiont. Therefore, this may profit from the nematode’s migration through the sulfide gradient that exists in the marine sediment (Ott et al. 1991). It has long been hypothesized that adult stilbonematids feed on their ectosymbionts. This is mainly because stable carbon isotope ratios of stilbonematids resemble those reported from free-living thiobacilli (Ott et al. 1991). Nevertheless, ectosymbiont ingestion has never been observed. Only the guts of *Leptonemella sp.* and *S. majum* were found to contain ectosymbiont-like bacteria displaying signs of lysis (Ott et al. 1991). However, apart from digestion, ectosymbiont-produced organic compounds could directly diffuse through the worm cuticle [see for example (Rutherford and Webster 1974)].

## An inventory of *L. oneistus* putative immunity pathways based on the *Caenorhabditis elegans*-pathogen model

The presence of a specific bacterial phylotype on the cuticle of each described stilbonematid species is exceptional in the nematode phylum. From the approximately 4,000 described marine nematode species (Miljutina et al. 2010) less than 2%—comprising members of the closely related families *Epsilonematidae*, *Draconematidae* and *Desmodoridae*—show at least occasional microbial epigrowth (J.A. Ott, unpublished observation). I hypothesize that *L. oneistus* and its ectosymbiont are adapted to each other in one of two, non-mutually exclusive, ways: (1) the ectosymbiont down-regulates host immunity and produces antimicrobials to protect the immunodepressed host from the attack of deleterious microbes; (2) the ectosymbiont is subject to a similar immune reaction that a pathogen would trigger, but does not succumb to it. In this last scenario the ectosymbiont may even exploit components of the host defense to (a) establish or strengthen its physical contact to the host or (b) regulate its proliferation in a way that facilitates contact persistence.

The immune system of the model nematode *Caenorhabditis elegans* was reviewed recently (Alper et al. 2007; Schulenburg et al. 2008; Irazoqui et al. 2010) (Ewbank and Zugasti 2011; Tan and Shapira 2011) (See Appendix and Fig. 2). Although *C. elegans* is a model organism for the study of host-microbe interactions, research to date has focused exclusively on interactions with pathogenic microbes. *C. elegans* is able to change its behavior in order to avoid deleterious microorganisms (Pradel et al. 2007). If this does not exempt it from their contact, *C. elegans* will rely on its epithelial immunity to respond to pathogens because it does not have circulating immune cells. Different microbes trigger distinct, specific immune reactions so that *C. elegans* can discern between non-pathogenic versus pathogenic, and also between different classes of microbes (See Appendix). The specificity of this custom made immune response may arise at the recognition level or at the effector level, but may also be achieved through differential immune regulation (e.g. different microbes cause a different degree of activation of one or more signaling pathway(s) or a different integration among pathways) (Schulenburg et al. 2008). Remarkably, *C. elegans* epithelial immunity does not rely on Toll-like receptors, despite their undisputed role in mammalian immunity (Pujol et al. 2001). Moreover, many genes encoding Toll-NF-kB pathway components are lacking not only from *C. elegans* but also from all the available nematode genomes (Irazoqui et al. 2010).

Epithelial immunity elicited in the epidermal cells differs from that in the intestinal cells. Although the p38 MAPK pathway (Kim et al. 2002) plays a central role in both epithelial tissues, two additional signaling pathways are integrated into it and are responsible for its neuronal modulation: the TGF-β-signaling pathway, in epidermal immunity, and the insulin/IGF-1 signaling (IIS) pathway, in intestinal immunity (Garsin et al. 2003) (Fig. 2).

### Epidermal immune defense

It is unclear if *L. oneistus* epidermal cells immunologically react to the presence of bacteria attached to the worm’s intact cuticle. Although an intact nematode cuticle may be “impermeable” to Microbe-Associated Molecular Patterns (MAMPs), there is a continuum between the GSO lumen and the nematode surface (see Section 1). Therefore, it is conceivable that the GSO gland cell(s) sense and respond to bacteria instead of, or in addition to, the epidermal cells. Moreover, it is also possible that secretion of immune effectors by each GSO may be locally modulated by its neuronal cell.

Adult *L. oneistus* express transcripts encoding for the p38 MAPK module components SEK-1 and PMK-1 and a TGF-β activated MAP3K protein (adult *L. oneistus* transcriptomic data are available at http://genepool.bio.ed.ac.uk/GP_Partigene/2008075_SilviaBulgheresi/; clusters encoding for orthologs of *C. elegans* immunity pathway components are listed in Table 1). Putative p38 MAPK module activators expressed in *L. oneistus* may include heteromeric G protein component β RACK-1 and protein kinase C PLC-3, as well as a Tribbles homolog 1 (*C. elegans* NIPI-3). As anticipated, the neuronally activated TGF-β pathway is integrated into the p38 MAPK pathway in the *C. elegans* epidermal immunity. *L. oneistus* expresses transcripts encoding for all the TGF-β pathway basic components: DBL-1, a TGF-β type I receptor, and Smad proteins 2, 3, 4 and 6. At the immune effector level, transcripts encoding for caenacin or immune-related neuropeptide-like proteins (NLPs) have not been found in the available transcriptomic data (See also Section 4 and Table 2).

**Table 1:** C. elegans immunity pathway components (adapted from Schulenburg and Boehnisch, 2008), *H. sapiens* orthologs, and *L. oneistus* clusters encoding for putative orthologs. Isogroup abundance indicates the number of sequences making up each cluster (when more than one cluster is present, the number of sequences making up each cluster were added and the total number is displayed); isogroup abundance may be directly correlated to transcript abundance. 454 transcriptomic data generated from symbiotic *L. oneistus* adults is available at: http://genepool.bio.ed.ac.uk/GP_Partigene/2008075_SilviaBulgheresi/; 14,747 putative genes (11,368 contigs and 3,379 singletons) corresponding to 5,888,206 bases were identified

*C. elegans*	*H. sapiens*	*L. oneistus* ortholog-encoding clusters	Isogroup abundance
TGF-beta pathway			
Neuron			
DBL-1	TGF--β ligand	LOP04675	3

Epithelial cell			
SMA-6	Type I TGF-β receptor	LOP07766	1
SMA-2	Smad protein	LOP03197	1
SMA-3	Smad protein	LOP03388	2
SMA-4	Smad protein	LOP06423	1

Insulin/IGF-1 (IIS) pathway			
Neuron			
GOA-1	G protein α subunit	LOP05008	2
DGK-1	Diacylglycerol kinase -β	LOP03176, LOP07534	2

Epithelial cell			
DAF-2	Insulin/IGF-1 receptor	LOP06243	1
AGE-1	Phosphatidylinositol 3-kinase	LOP04446, LOP08838, LOP07119	3
AKT-1/AKT-2	Rac Ser/Thr protein kinase	LOP05995, LOP09110, LOP06152	5
SGK-1	Serum/glucocorticoid regulated kinase 1	LOP05995, LOP09110	3
DAF-16	FOXO family transcription factor	LOP10192, LOP07765	5

P38 MAPK pathway			
Epidermal cell			
RACK-1	G protein β subunit	LOP01842	9
PLC-3	Phospholipase C γ	LOP05069	2
PKC-3	Protein kinase C iota type	LOP11897	2

Intestinal cell			
EGL-30^b^	G protein G(q) α subunit	LOP06585, LOP05008	4
DKF-2	Ser/Thr protein kinase D	LOP07419, LOP10773	4
NSY-1	ASK1 MAPKKK	LOP04342^a^, LOP09080^a^	5
SEK-1	MKK3, MKK6 MAPKK	LOP05076, LOP05850, LOP07733, LOP02761	7
PMK-1	p38 MAPK	LOP01689, LOP03611, LOP07256	13

^a^No NSY hortologue was identified; clusters encode for a ortholog of human TGF-beta activated kinase MAPKKK7

^b^In *C. elegans* the Gqα signaling component EGL-30 is also expressed in the neuron and can modulate intestinal immunity

**Table 2:** *L. oneistus* clusters encoding for putative orthologs of *C. elegans* immune effectors. See Table 1 legend for explanation of isogroup abundance and information about the used transcriptomic data

*C. elegans*	*L. oneistus* ortholog-encoding clusters	Isogroup abundance
Caenopores (saposin-like proteins; SPPs)		
SPP-10	LOP01886,LOP01470,LOP01471	72

Lysozymes		
LYS-8	LOP02195	18
LYS-5	LOP03710	6
LYS-6	LOP07511,LOP06316	5
LYS-10	LOP11094	3
LYS-4	LOP03904	2

C type lectin domain-containing proteins (CTLD)		
CLEC-50	LOP07024,LOP02441,LOP00362,LOP00364,LOP00366,LOP00367,LOP00371,LOP00372,LOP00373,LOP00376,LOP00368,LOP00369,LOP00375,LOP00377,LOP02333,LOP00378,LOP00379,LOP00380,LOP00381,LOP01037,LOP01038	442
CLEC-178	LOP04079,LOP01041,LOP01042	78
CLEC-48	LOP05939,LOP02725	18
CLEC-56	LOP01721	4
CLEC-11	LOP07307	2
CLEC-3	LOP03907	2
CLEC-150	LOP07069	2
CLEC-10	LOP05031	1

FIPR proteins		
FIP-1-like	LOP04451	4

Glycine-rich proteins		
Glycine-rich protein	LOP00858,LOP00856	22

### Intestinal immune defense

Apart from covering its surface, it is possible that the ectosymbiont may, at some *L. oneistus* developmental stage, proliferate and persist in its gut. As already mentioned above, the pharynx of the stilbonematids is weak and, in contrast to *C. elegans*, they do not possess an efficient grinder-like organ, which can efficiently triturate the bacteria. In *L. oneistus*, the gene encoding for protein kinase DFK-2 is transcribed and this could activate the p38 MAPK module in intestinal cells. Additionally, the IIS pathway might play a role in mediating microbial recognition in the gut. Although we did not identify transcripts encoding for neuronally secreted insulin-like ligands such as INS-7 (see Appendix), those encoding for orthologs of the insulin/IGF-1 receptor DAF-2, the Ser/Thr kinases AKT-1/AKT-2/SGK-1 and DAF-16 were all present. The stress protective transcription factor DAF-16 was long hypothesized to be the only capable to confer pathogen resistance to *C. elegans* (see Section 4 and Table 2 for putative *L. oneistus* immune effectors expressed in intestinal immune response).

## An inventory of putative *L. oneistus* immune effectors based on the *C. elegans*-pathogen model

In *C. elegans* the activation of immune pathways leads to the production and secretion of the following antimicrobial proteins and peptides (Ewbank and Zugasti 2011): caenopores (or SPPs), lysozymes, lectins, antibacterial factors (ABF) peptides, NLPs, caenacins, fungus-induced proteins (FIPs), FIP-related proteins (FIPRs) and glycine-rich secreted proteins (GRSPs). Of these, ABFs are constitutively expressed and the last five classes are epidermally expressed.

At least 33 *C. elegans* SPPs were identified (Roeder et al. 2010). They are pore-forming proteins structurally and functionally similar to vertebrate granulysin and NK-lysin and *L. oneistus* express one of them, the spp-10 ortholog.

Lysozymes are thought to be secreted in the *C. elegans* intestinal lumen where they may directly act on both Gram-negative and positive bacteria (Mallo et al. 2002; O’Rourke et al. 2006; Schulenburg et al. 2008). Of the 15 known in *C. elegans*, five putative orthologs are expressed by *L. oneistus* (lys-4,-5,-6,-8, and 10). *C. elegans lys-8*—which might be the most abundantly expressed in *L. oneistus*—is up-regulated in the intestine upon infection by the Gammaproteobacterium *Serratia marcescens* (Mallo et al. 2002).

*C. elegans* lectins might (a) bind microbial surface sugars and be involved in their recognition, (b) make microbes more susceptible to be phagocytosed by immune cells, (c) have antimicrobial activity, and/or (d) mask host epitopes targeted by microbial effectors (Schulenburg et al. 2008). The regulation of their expression is very complex, microbe-dependent and controlled by several signal transduction pathways (Mallo et al. 2002; O’Rourke et al. 2006; Alper et al. 2007). Among the lectins, 278 CTLD proteins were identified, with the most numerous class (class I) being characterized by a single CTLD (141 proteins, 99 of which are secreted (Schulenburg et al. 2008)). Other classes carry additional CTLDs, CUB domains, a *Caenorhabditis*-specific CW domain, von Willebrand factors (VWA), or a proline-rich repeat (PRR). Each CTLD protein class contains pathogen-induced genes and silencing of *clec-17*, *clec-60*, and *clec-86* shows a direct role in immune defense (O’Rourke et al. 2006).

*L. oneistus* express orthologs of eight *C. elegans* CTLD proteins. In particular, a *clec-50*-like gene appears to be very abundantly expressed (442 isogroups grouped in 21 clusters). As observed for *lys-8*, *C. elegans clec-50* is up-regulated in the intestine upon *S. marcescens* infection (Mallo et al. 2002). The *C. elegans* CTLD most similar to Mermaid is CLEC-178. Although the *L. oneistus clec-178* ortholog appears to be abundantly expressed, *mermaid* transcripts are absent from the transcriptomic data and only present in a previously generated EST dataset (http://www.nematodes.org/NeglectedGenomes/NEMATODA/Laxus_oneistus/index.html). One possible explanation for this very surprising finding is that *mermaid* genes are not constantly and abundantly expressed in the adult *L. oneistus*. Given that the Mermaid proteins appear to be very stable (Bulgheresi et al., 2006) transcription of their corresponding genes in the P GSOs may be limited to particular developmental stages, those in which the ectosymbiosis must be established. This would also explain why the ectosymbiont is not able to recolonize bacteria-free areas artificially produced on adult nematodes (Hentschel et al. 1999).

Other potential immune effectors conserved in both *C. elegans* and *L. oneistus* are FIPRs and GRSPs. We did not identify *L. oneistus* transcripts encoding for orthologs of *C. elegans* antibacterial factors (ABFs), immune-related NLPs or caenacins. In *C. elegans*, the first are mostly effective against Gram+, whereas the expression of NLPs and caenacins is triggered by cuticle-infecting fungi, but not by bacteria.

## Concluding remarks

Based on the available *L. oneistus* transcriptomic data and by using the *C. elegans*-pathogen model system as a reference, a few preliminary conclusions can be drawn: 1) *L. oneistus* constitutively express the signaling pathway components necessary for these nematodes to react to the presence of its ectosymbiont or to other microbes. In particular, transcripts encoding for all the members of the TGF-β pathway, which is central in *C. elegans* epidermal immunity, are present. 2) More conservation seems to exist among signaling pathways working in *C. elegans* and *L. oneistus* than among the downstream effectors that they regulate. This is not surprising as antimicrobial compounds are notorious for being poorly conserved. 3) *L. oneistus* appear to constitutively express immune effector genes, which are up-regulated by *C. elegans* upon neuronal activation of the TGF-β pathway by the Gammaprotebacterium *Serratia marcescens* (such as *clec-50* and *lys-8*); it is conceivable that *L. oneistus* clec-50 and lys-8 orthologs are expressed in response to the (gammaproteobacterial) ectosymbiont. 4) The *L. oneistus* GSOs underlie both P and A cuticle. Therefore, throughout the A-P axis, the presence/absence of bacteria could trigger differential secretion of immune effectors by A and P GSOs. This is possible, given that each GSO may secrete immune effectors in response to a local signal. The establishment of gene silencing in *L. oneistus* is necessary to prove these conclusions. At the same time, understanding the molecular basis of *C. elegans* interaction with non pathogenic microbes would help elucidate the molecular base of nematode-bacterium symbioses.

## Appendix. Epidermal and intestinal immunity in *C. elegans*

A few pathogenic bacteria and fungi are known to infect *C. elegans* by adhering to its cuticle (Zugasti and Ewbank 2009). *D. coniospora* attach to- and pierce the nematode cuticle. After penetrating the epidermis, hyphae then develop and grow inside the host, eventually killing it (Dijksterhuis et al. 1990). In the case of p38 MAPK-mediated epidermal immunity, the binding of an unknown ligand to an unknown receptor results in successive activation of heterotrimeric G protein, protein kinase (s) C (such as PKC-3) and the p38 MAPK module (NSY-1/SEK-1/PMK-1). Activation of the module results in the expression of antimicrobial peptides such as the neuropeptide-like peptide (NLP; see Section 4) nlp-29. Neuronally secreted DBL-1 may also trigger epidermal immunity. The identity of the DBL-1 secreting neurons is unknown. In this case, DBL-1 receptor-regulated Smad proteins (Sma-6, 2, -3, and -4) would activate (an) unknown transcription factor(s) in the epidermal cell and these factors, in turn, would switch on expression of the caenacin (see Section 4) cnc-2.

Most bacterial pathogens colonize the intestine of *C. elegans* and affect defense gene expression in its gut. In intestinal defense, the p38 MAPK cassette can be activated by the protein kinase DKF-2 and it regulates the expression of immunity genes such as the lysozyme encoding *lys-2* (see Section 4). As it is the case for epidermal immunity, we do not know the ligand(s) and the G protein-coupled receptor(s) leading to DFK-2 activation. In addition to the p38 MAPK pathway, the IIS pathway functions in intestinal immunity. This is a very conserved regulator of metabolism, stress resistance and ageing, with increasingly recognized roles in metazoan immune homeostasis (Peng 2010) (Becker et al. 2010). The activation of the IIS pathway in the intestinal cell is controlled by INS-7, a ligand of the insulin/IGF-1 receptor DAF-2 (Kawli and Tan 2008). The levels of neuronally secreted INS-7 are in turn regulated by both Goα signaling, which includes EGL-30 and EGL-8 (see Table 1) and Gqα signaling, which includes GOA-1 and DGK-1 (see Table 1). If INS-7 is abundant, DAF-2 activation triggers a phosphorylation cascade involving the phosphatidylinositol 3-kinase protein AGE-1, and lipid and serine/threonine kinases (AKT-1/AKT-2/SGK-1). These phosphorylation events lead to the phosphorylation and cytoplasmic retention of the FoxO forkhead transcription factor homologue DAF-16. Cytoplasmic DAF-16 can not ignite intestinal cell immune defense. On the other hand, if INS-7 secretion and, consequently, DAF-2 function is reduced, DAF-16 is translocated into the nucleus and this triggers the expression of antimicrobial genes, such as the intestinal lysozyme LYS-7 and the saposin-like protein SPP-1 (lysozymes and SPPs are discussed in Section 4).
